# Predicting cancer relapse following lung stereotactic radiotherapy: an external validation study using real-world evidence

**DOI:** 10.3389/fonc.2023.1156389

**Published:** 2023-07-12

**Authors:** Angela Davey, Maria Thor, Marcel van Herk, Corinne Faivre-Finn, Andreas Rimner, Joseph O. Deasy, Alan McWilliam

**Affiliations:** ^1^ Division of Cancer Sciences, School of Medical Sciences, Faculty of Biology, Medicine and Health, The University of Manchester, Manchester, United Kingdom; ^2^ Department of Medical Physics, Memorial Sloan Kettering Cancer Center, New York, NY, United States; ^3^ Department of Clinical Oncology, The Christie NHS Foundation Trust, Manchester, United Kingdom; ^4^ Department of Radiation Oncology, Memorial Sloan Kettering Cancer Center, New York, NY, United States

**Keywords:** image-based data mining, real world data, biomarker-by-treatment interactions, local relapse, NSCLC, stereotactic ablative body radiotherapy (SABR), personalized medicine, external validation

## Abstract

**Purpose:**

For patients receiving lung stereotactic ablative radiotherapy (SABR), evidence suggests that high peritumor density predicts an increased risk of microscopic disease (MDE) and local-regional failure, but only if there is low or heterogenous *incidental* dose surrounding the tumor (GTV). A data-mining method (*Cox-per-radius*) has been developed to investigate this dose-density interaction. We apply the method to predict local relapse (LR) and regional failure (RF) in patients with non-small cell lung cancer.

**Methods:**

199 patients treated in a routine setting were collated from a single institution for training, and 76 patients from an external institution for validation. Three density metrics (mean, 90^th^ percentile, standard deviation (SD)) were studied in 1mm annuli between 0.5cm inside and 2cm outside the GTV boundary. Dose SD and fraction of volume receiving less than 30Gy were studied in annuli 0.5-2cm outside the GTV to describe *incidental* MDE dosage. Heat-maps were created that correlate with changes in LR and RF rates due to the interaction between dose heterogeneity and density at each distance combination. Regions of significant improvement were studied in Cox proportional hazards models, and explored with and without re-fitting in external data. Correlations between the dose component of the interaction and common dose metrics were reported.

**Results:**

Local relapse occurred at a rate of 6.5% in the training cohort, and 18% in the validation cohort, which included larger and more centrally located tumors. High peritumor density in combination with high dose variability (0.5 - 1.6cm) predicts LR. No interactions predicted RF. The LR interaction improved the predictive ability compared to using clinical variables alone (optimism-adjusted C-index; 0.82 vs 0.76). Re-fitting model coefficients in external data confirmed the importance of this interaction (C-index; 0.86 vs 0.76). Dose variability in the 0.5-1.6 cm annular region strongly correlates with heterogeneity inside the target volume (SD; ρ = 0.53 training, ρ = 0.65 validation).

**Conclusion:**

In these real-world cohorts, the combination of relatively high peritumor density and high dose variability predicts increase in LR, but not RF, following lung SABR. This external validation justifies potential use of the model to increase low-dose CTV margins for high-risk patients.

## 1 Introduction

Patients with early-stage lung cancer who are medically inoperable or refuse surgery will receive stereotactic body radiotherapy (SABR) as standard of care (1). It is a well-tolerated and successful treatment, with five-year local relapse (LR), regional failure (RF), and distant metastasis (DM) rates at ranges between 8-11%, 10-13%, and 11-22% respectively (2–5). SABR is characterized by high dose radiation delivered by multiple conformal beams to precisely target the tumor and avoid surrounding tissue. To maintain a conformal dose distribution, dose heterogeneity inside the planning target volume (PTV) is permitted (6). It is still unknown what part of the tumoral dose distribution is affecting tumor control the most (7), and further considerable institutional differences exist in treatment planning approaches (8). Better understanding of the level of tumor dose homogeneity/heterogeneity could lead to changes in radiotherapy planning to improve patient outcomes. In some situations, data from real-world settings allows us to test hypotheses on the impact of changes that can be made to the treatment planning process as an alternative to costly clinical trials. Real word data also has the advantage of being more inclusive of a general population. This is particularly relevant in the context of patients with lung cancer treated with SABR as most of them are elderly, frail and with multiple comorbidities. These patients are typically excluded or underrepresented in clinical trials (9).

The association between dose and LR has been well investigated through the link between the prescription dose and tumor control probability. Such studies report dose associations with the isodose surface encompassing the PTV (10, 11), the isocenter (12) and the average of the two (13). A study on 1500 patients emphasized the importance of ensuring high doses within the gross tumor volume (GTV) to promote local control (13). Reports have also detailed the importance of high dose *outside* the GTV as means of treating microscopic disease (MDE) and nodal micro-metastases that could be responsible for treatment failure (14, 15). Further studying this effect may identify associations that can be utilized to personalize treatments for patients at *high-risk* of failure.

Assessing pre-treatment imaging biomarkers is a non-invasive approach to identify high-risk patients who could be candidates for treatment adaptation. Salguero et al. demonstrated that CT-based GTV circularity and surface density predict high-risk of MDE (14). High-risk of MDE also translated into an increased risk of local-regional failure if patients had low dose within 1.5cm from the GTV (16). Previous efforts have, however, focused on using tumor dose parameterizations, histological, or clinical characteristics alone to study and stratify risk of MDE (10–13, 17).

Ignoring risk stratification can lead to incorrectly claiming a lack of association (18). In our previous work, we developed a *Cox-per-radius* method to investigate the interaction between imaging density biomarkers and dose in independent annuli surrounding the GTV (19). In that study we demonstrated that the interaction between CT-based imaging biomarkers and dose far outside the tumor is linked to DM. In this study, we will use our previously developed method to assess whether a similar associations can be identified for LR and RF using two real-world patients cohorts. We will utilize real-world data from geographically separate institutions to explore the generalizability of any identified patterns.

## 2 Methods

### 2.1 Clinical data and patient follow-up

#### 2.1.1 Training data

Data was available for 195 patients with T1-2 N0M0 non-small cell lung cancer (confirmed histologically or suspected based on radiology) who were treated with SABR for primary lung cancer during 2011-2017 at The Christie NHS Foundation Trust, with 60 Gy in 5 fractions on consecutive weekdays. Institutional approval was granted to collect and analyse this data (REC reference: 17/NW/0060). All patients were staged with both a CT and 18F-FDG positron emission tomography (PET) scan, but did not always receive a histological diagnosis. Four-dimensional computed tomography scans (4D-CT) and three-dimensional dose distributions were available, as described previously and in **Supplementary Material** (SM), Section 1 (19). Clinical data was retrospectively collected from structured e-forms completed in routine practice. Clinical data was available on tumour lobe location, age, sex, Eastern Cooperative Oncology Group (ECOG) performance status (functional ability), ACE27 comorbidity score (describing the presence and severity of existing medical conditions), and histological sub-type. Where available, histological sub-type diagnosis was classified as ‘adenocarcinoma not otherwise specified (ADC NOS)’, ‘squamous cell carcinoma (SCC)’, ‘carcinoma NOS’ or other. Information on tumor centrality was not available.

In routine practice, patients were followed-up every three months for the first year, and six monthly thereafter. At the discretion of the clinician, a free-breathing CT is performed, PET or biopsy recommended when treatment failure is suspected. Recorded data on treatment failure was retrospectively collected from electronic records. Local relapse (LR) was defined as progression in or adjacent to the original treatment volume, based on clinical interpretation of ‘adjacent’ following reported definitions as guidance (20). Regional failure (RF) was defined as recurrence in regional lymph nodes (hilar or mediastinal). Time to failure was recorded from the start of radiotherapy to the date of the first scan that showed progression. Patients were censored at the most recent follow-up in the absence of failure.

#### 2.1.2 Validation data

For validation, data were available for 139 patients with T1-2 NOM0 early-stage NSCLC treated with a range of fractionation regimes (see SM, **Table 1**) treated at Memorial Sloan Kettering Cancer Center (MSKCC) between 2014 and 2017. There was no agreement in treatment schedules between the training and validation data. For validation, we limited selection to patients treated with 50Gy in 5 fractions treated on consecutive weekdays, as the most common treatment schedule, and included all such patients treated with that regime during this time frame. All patients included had been staged with a CT and PET scan. A data-sharing agreement was in place and a study analysis plan made available online at the start of the collaboration (21). Clinical data was collected retrospectively from structured e-forms completed in routine practice. Clinical data was available on tumor lobe location, age, sex, Karnofsky performance status (a rating of 0-100 measuring a patient’s ability to perform daily tasks), and histological sub-type (SCC, ADC, or other). Information on comorbidity score or tumor centrality was not available. The Karnofsky performance status was scaled to the ECOG gradings to match the training cohort using a published guide (22).

**Table 1:** A table to demonstrate patient demographic differences in training and validation data.

**Characteristic**	**Training, N = 195^1^**	**Validation, N = 76^1^**	**p-value^2^**
**Tumor volume (cc)**	4 (0 - 31)	14 (0 - 158)	<0.001
**Tumor motion (cm)**	0.56 (0.00 - 3.43)	0.36 (0.00 - 2.73)	0.016
**Tumor lobe location**			0.7
Lower	69 (35%)	29 (38%)	
Upper	126 (65%)	47 (62%)	
**Age (years)**	75 (45 - 92)	78 (52 - 92)	0.036
**Biological sex**			0.3
Female	99 (51%)	44 (58%)	
Male	96 (49%)	32 (42%)	
**Histological subtype**			<0.001
Adenocarcinoma, NOS	35 (37%)	45 (59%)	
Squamous cell carcinoma	37 (39%)	24 (32%)	
Carcinoma, NOS	14 (15%)	0 (0%)	
Other	8 (8.5%)	7 (9.2%)	
Unknown	101	0	
**Performance status (ECOG)**			<0.001
0	3 (1.8%)	29 (38%)	
1	64 (37%)	43 (57%)	
2	83 (49%)	4 (5.3%)	
3	21 (12%)	0 (0%)	
Unknown	24	0	
**Local relapse**	13 (6.7%)	12 (16%)	0.020
**Regional failure**	15 (7.7%)	5 (6.6%)	0.8

^1^Statistics presented: n (%); Median (range).

^2^Wilcoxon rank sum test; Pearson’s Chi-squared test; Fisher’s exact test.

Tumours were larger and less mobile in the validation data compared to training. Worse performance status was reported in the training set. Differences were also identified in histological sub-type with a larger proportion of adenocarcinoma in the validation data, but this could be influenced by the missing data reported for training. In the validation set there were significantly more local relapses but a similar percentage of regional failures.

Data was also collected on recurrences for this cohort. Local relapse was defined as new tumor growth at the site of prior CT; and recurrences were typically confirmed by PET (to demonstrate local FDG avidity) and/or biopsy. Regional failure was defined as recurrence in regional lymph nodes.

### 2.2 Imaging and dosimetric data

Both training and validation cohorts had treatment plans that included a ‘motion-adapted’ GTV (iGTV) which incorporates the GTV combined across all respiratory phases. In both institutions, this is outlined on the maximum intensity projection (MIP) and edited on individual respiratory phases. The PTV was also recorded, which represents the iGTV plus a 5mm expansion (with a 2-3mm additional CTV in the validation cohort). To extract the tumor volume (GTV) for every phase, an in-house process was applied to estimate and remove the motion adaptation using rigid tumor registration across 4D phases (23). As a result, a GTV contour was available for every phase and two additional clinical variables recorded: tumor volume and tumor motion amplitude. Results of the registration were assessed visually on a movie-loop of registered phases, and all GTV contours were visually approved by a single observer.

Three-dimensional dose distributions were available for all patients and were converted to biologically equivalent doses in 2Gy fractions (EQD2) using α/β=10. Independently, the same set of distributions were blurred according to respiratory motion (derived from the registration above) and then converted to EQD2 – which represents the *blurred* dose represented on a reference phase (for which we used the middle, i.e., 50% phase). The mean, maximum, minimum, and standard deviation (SD) of the dose inside the PTV was calculated in both cohorts based on the planned dose. The *blurred* dose provides an indication of the *planned* tumor dose over the respiratory cycle. From this distribution, the mean, maximum, minimum, and SD dose on the generated GTV was calculated.

#### 2.2.1 Density metrics

The radial histogram framework described in our previous work was implemented to measure density and dose at radial distances from the generated GTV for all patients (19). A 2D cross-histogram of density vs distance in bins of 1mm annuli from -0.5 to 2cm from the GTV was formed for each 4D phase considering lung tissue only. Only lung tissue was considered for density metrics so that higher density is not just a surrogate for nearby organs-at-risk. For each patient, a single phase was selected as the most stable compared to neighboring phases and used for density analysis in the remaining analysis (as detailed in previous work) (19, 24). Summary curves were then extracted from the 2D histogram for each patient to describe the mean, 90^th^ percentile, and SD density at the defined distances from the GTV. The summary curves were smoothed with Gaussian smoothing (σ = 1.5mm) (as annuli thickness is less than slice thickness) and stored for each patient.

#### 2.2.2 Dose metrics

To extract dosimetric information, scans were cropped based on the body contour, and dose-volume histograms were extracted in 1mm annuli from the *blurred* dose distribution in the region 0.5-4cm radially from the GTV on the reference phase (19). Smoothed curves were summarized by dose SD, and the fraction of volume in each 1mm rim that receives EQD2 of less than 30Gy, which is a dose threshold reported for controlling MDE (25). An additional curve of mean dose was extracted for visualization only.

#### 2.2.3 Exploratory comparison

The average curve across patients for each metric as function of radius was calculated in training and validation. The curves did not inform selection of which variables to use in the remaining analysis but assist in interpretating the results. We visually assessed the images for all patients to note any qualitative characteristics that may lead to differences between the two groups.

### 2.3 Model development

#### 2.3.1 Clinical model and ‘standard’ dose metrics

For training, a reference Cox model was derived for both LR and RF including clinical variables with complete data for all patients – which were tumor lobe location, age, sex, tumor motion amplitude, and tumor volume. The concordance index (C-index), Akaike Information Criterion (AIC), and any variables that statistically significantly predicted LR and/or RF were recorded. Each GTV and PTV dose-related parameter extracted in Section 2.2 was then included in the Cox model individually to determine whether it was associated with RF and LR, and a likelihood-ratio test was performed for models with and without the dose parameter to determine if there was a significant improvement in model performance.

#### 2.3.2 Interaction maps

The *Cox-per-radius* method was applied for each outcome separately (LR and RF); full details reported in previous work (19). Briefly, for each combination of dose and density in independent annuli, the density feature, dose metric, and interaction term (density*dose) were added to the clinical model. A likelihood-ratio test was performed for models with and without the interaction to produce a heat-map of p-values describing the benefit of the interaction for prediction in each dose-density annulus combination. Regions of statistical significance (defined by p<0.05) were highlighted on the heat-map.

Region size post-processing was then used as multiple-testing correction on the heat-maps to ensure regions were truly not less than 3mm thickness in either the dose or density scale (likely representing spurious associations) (26, 27). The average height and width of each region defined boxes on a heat-map that represent the significant distances for dose and density independently. From the defined independent annuli, the dose and density values in the identified regions were extracted and included in overall Cox models. Internal validation was performed over 500 bootstrap resamples to estimate the interaction coefficient stability and model performance. A secondary post-processing step was then performed, which removed unstable interaction coefficients on internal validation or interactions that did not improve the performance of the clinical model (C-index). Unstable coefficients are those that show both a negative and positive effect within the 95% confidence interval across resamples.

#### 2.3.3 Final models, internal validation, and interpretation

Coefficients and p-values were reported for models with and without the interaction term. To interpret the direction and size of the association between density and outcome, *contrast plots* were created with log(hazard ratio) on the y-axis and different density values on the x-axis for the 10^th^ percentile, median, and 90^th^ percentile value of the dose parameter in the relevant region (28).

Based on the results from the internal validation, the concordance-index was adjusted for optimism. For each bootstrapped model we calculated an estimate of the C-index and calculated the difference between the C-index of the model in the bootstrapped data and in the original data. To calculate the optimism-adjusted C-index, the median difference across all resamples was subtracted from the original C-index. This internal validation was performed for the clinical model, and the new models with and without an interaction term for comparison. For all models, the median and 95% confidence interval of the C-index across the bootstrap resamples were recorded.

For interpretation of the identified dose location, correlations were investigated between the relevant dose parameter and the metrics extracted in Section 2.2.

### 2.4 External validation

No formal advice is currently reported for external *‘validation’* in an image-based data mining framework. Following the stricter advice of Royston et al. on validation in prediction modelling (29), the model coefficients developed on the training data were applied in the new data-set to build a validation Cox model. The C-index of the validation model was recorded and compared to that identified from the internal validation. This process was performed for the clinical model and repeated for the new models with and without the interaction terms.

As a second comparison (as opposed to strict prediction-model validation) we re-fit the model coefficients in the new data set to determine if the significance of the interaction would still be identified. Contrast plots were created for this interaction, and the same correlations between other dose metrics performed for interpretation. As a further validation step, we also studied the stratification of patients with Kaplan-Meier plots in both training and validation using an arbitrary cut off for the median peritumour density, and the lowest and highest third of the identified dose metrics.

## 3 Results

### 3.1 Clinical, imaging and dosimetric data

For training, 195 patients with complete clinical, image and dose data were available (19). Thirteen patients had LR (6.5%) and 15 patients (8%) had RF. Complete clinical data was available on tumor lobe location, age, sex, tumor motion amplitude, and tumor volume. In the training data, we report 52% missing data on histological sub-type and 12% on performance status – hence these were excluded from further analysis.

In the validation set, we first selected patients who were treated with the same number of fractions as the training data-set, this left 95 patients treated with 50Gy in 5 fractions. Out of the remaining patients ten more were excluded due to: missing radiotherapy data (n = 1), missing 4D-CT phases (n = 5), alternative planning approach (breath-hold; n = 2), or no iGTV (n = 2). On visual assessment of the GTV generation, we observed seven failures related to registration and three related to segmentation. Overall, 76 patients were available for analysis with complete data, and the difference between the training and the validation cohort are shown in **Table 1**. Twelve patients had LR (16%) and five RF (6.6%). In the validation cohort, the tumours were larger with a mean tumour volume of 14cc compared to 4cc, and a maximum volume of 158cc compared to 31cc. The tumours in the validation cohort also were more centrally located on visual assessment, which contributed to a significantly lower tumour motion amplitude. In the validation data, a larger distribution of adenocarcinoma compared to squamous cell carcinoma was recorded, and patients had better performance status.

### 3.2 Clinical models for training data

Firstly, using the training data, multivariable Cox models were built to predict LR and RF containing all clinical variables in **Table 1**, and the full tables are reported in SM **Table 2**. The summary of these models in **Table 2** demonstrates that only tumor volume is a prognostic factor for LR, and there are no prognostic factors for RF. Including ‘standard’ dose metrics in each model did not significantly improve model performance (SM, **Table 3**).

**Table 2:** Clinical model for local and regional failure in training data with significant variables, C-index, and Akaike Information Criterion (AIC) reported. Performance metrics were evaluated on the full data-set.

Outcome	Multivariable prognostic factors (p<=0.05)	C-index	AIC
**Regional**	None	0.74	142.3
**Local**	ln(Tumour volume) *(HR = 3.05, p = 0.006)*	0.79	100.0

**Table 3:** Concordance index of models on training and validation data.

**Model C-index**	**Training**	**Optimism-adjusted**	**Validation** **(without refitting)**	**Validation** **(with refitting)**
*Clinical*	0.81 *(0.66-0.94)*	0.76 *(0.61-0.80)*	0.58 *(0.37–0.76)*	0.76 *(0.58–0.91)*
*Clinical + SD dose + 90^th^ percentile density*	0.84 *(0.71-0.95)*	0.77 *(0.61-0.82)*	0.64 *(0.37-0.80)*	0.81 *(0.66-0.99)*
*Clinical + SD dose + 90^th^ percentile density* + SD dose*90^th^ percentile density	0.88 *(0.79-0.99)*	0.82 *(0.69-0.93)*	0.56 *(0.41-0.70)*	0.86 *(0.71-1.00)*

Optimism-adjusted results represent the internally validated models on the training data. Validation without re-fitting represents a strict prediction model validation procedure, whereas with re-fitting is building a new model with all the identified variables in the training data for comparison.

### 3.3 Radial dose and density metrics

**Figure 1:**
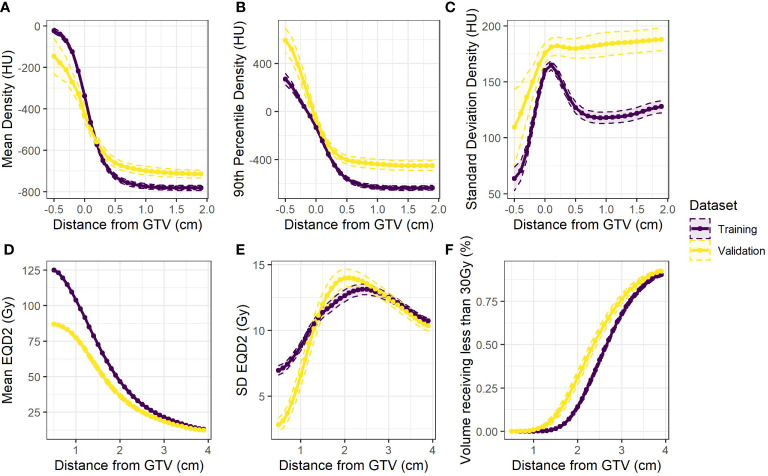
Population averages (dashed 95% confidence intervals) for the extracted variables against distance from the GTV for the training data (purple) and the validation data (yellow) **(A)** mean **(B)** 90^th^ percentile and **(C)** standard deviation of density. Also **(D)** mean and **(E)** standard deviation of equivalent dose, and **(F)** the fraction of volume in each rim receiving less than 30Gy.

Three density metrics (mean, 90^th^ percentile, SD) and three dose metrics (mean, SD, fraction volume receiving less than 30Gy) were extracted from both training and validation cohorts. These metrics were averaged across all patients and the differences visualised between cohorts as shown in **Figure 1**. An increased density heterogeneity inside and outside the tumour in the validation data was observed. The mean dose is lower in the validation cohort as the total prescription was 50Gy in 5 fractions compared to 60Gy in 5 fractions delivered in the training cohort. Differences in the other curves (**Figure 1**) include variation in both dose and density heterogeneity which likely represent institutional planning differences.

### 3.4 Dose-density interaction maps

Using the dose and density curves extracted for all patients, six *Cox-per-radius* maps were produced from combinations of the three density metrics and two dose metrics (SD, fraction volume receiving less than 30Gy) extracted for this stage of the analysis.

**Figure 2:**
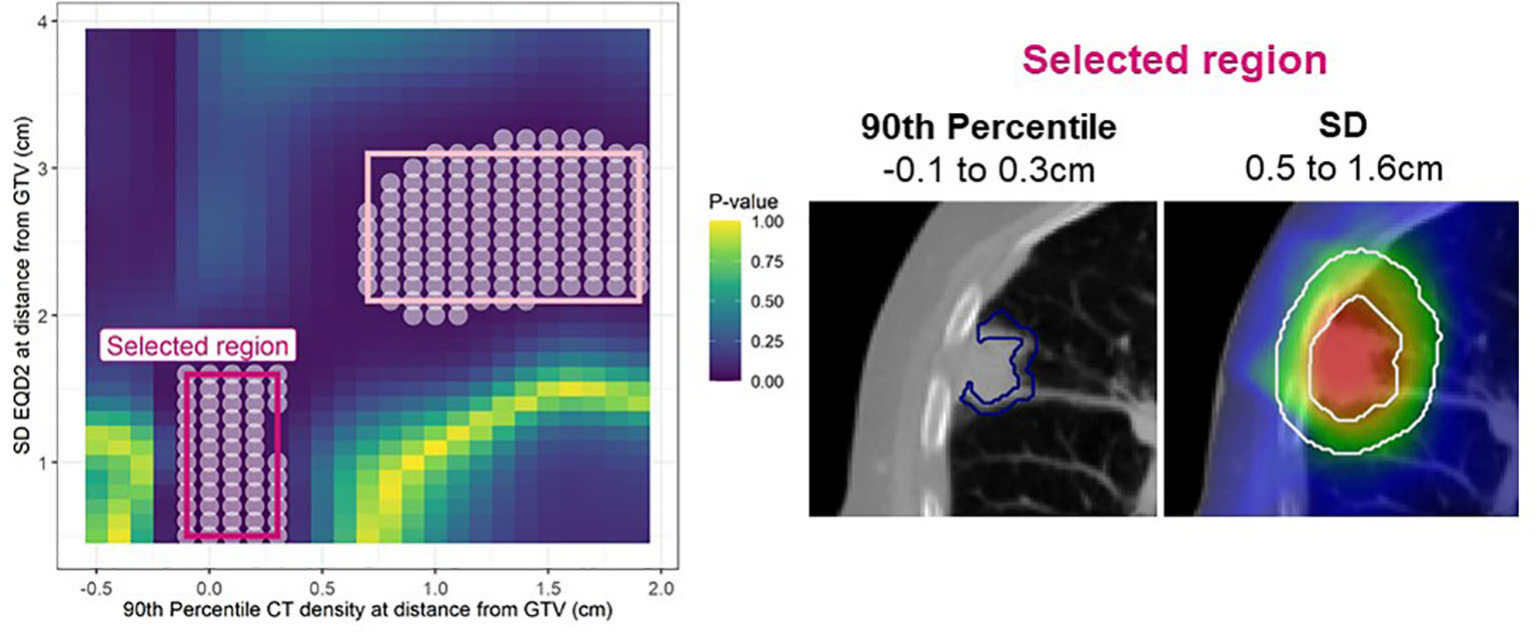
Left: Cox per radius significance map of interaction between 90^th^ percentile density vs SD EQD2 at distance from the GTV. The p-value reflects a likelihood-ratio test of improvement in model performance due to inclusion of the interaction between dose and density at each location. All significant points are shown with a white circle and the regions extracted for assessment in bootstrap are highlighted in pink. After post-processing only one region remained (bright pink). Right: The annuli volumes defined by the selected regions overlayed on an example patient.

In the analysis of LR, post-processing 1-40% of significant pixels were removed (SM, **Figure 1** and 2). After this, up to nine candidate regions were identified on each map. Of these nine regions, three were below the size threshold, one had unstable coefficients on internal validation, and four did not improve the clinical model performance (SM, **Table 3**), therefore only one consistently identified region remained (**Figure 2**).

This region indicates that 90^th^ percentile density at the peritumor border (-0.1 to 0.3cm) interacts with the dose SD 0.5-1.6cm and jointly predict LR. The interaction term was significant in 74% of bootstrap resamples and the interaction model performance had a median of 0.81 (95% confidence interval: 0.70 - 0.85) on internal validation. The larger region (light pink border; **Figure 2**) was not considered important as it did not improve the clinical model performance with a median C-index of 0.79.

**Figure 3:**
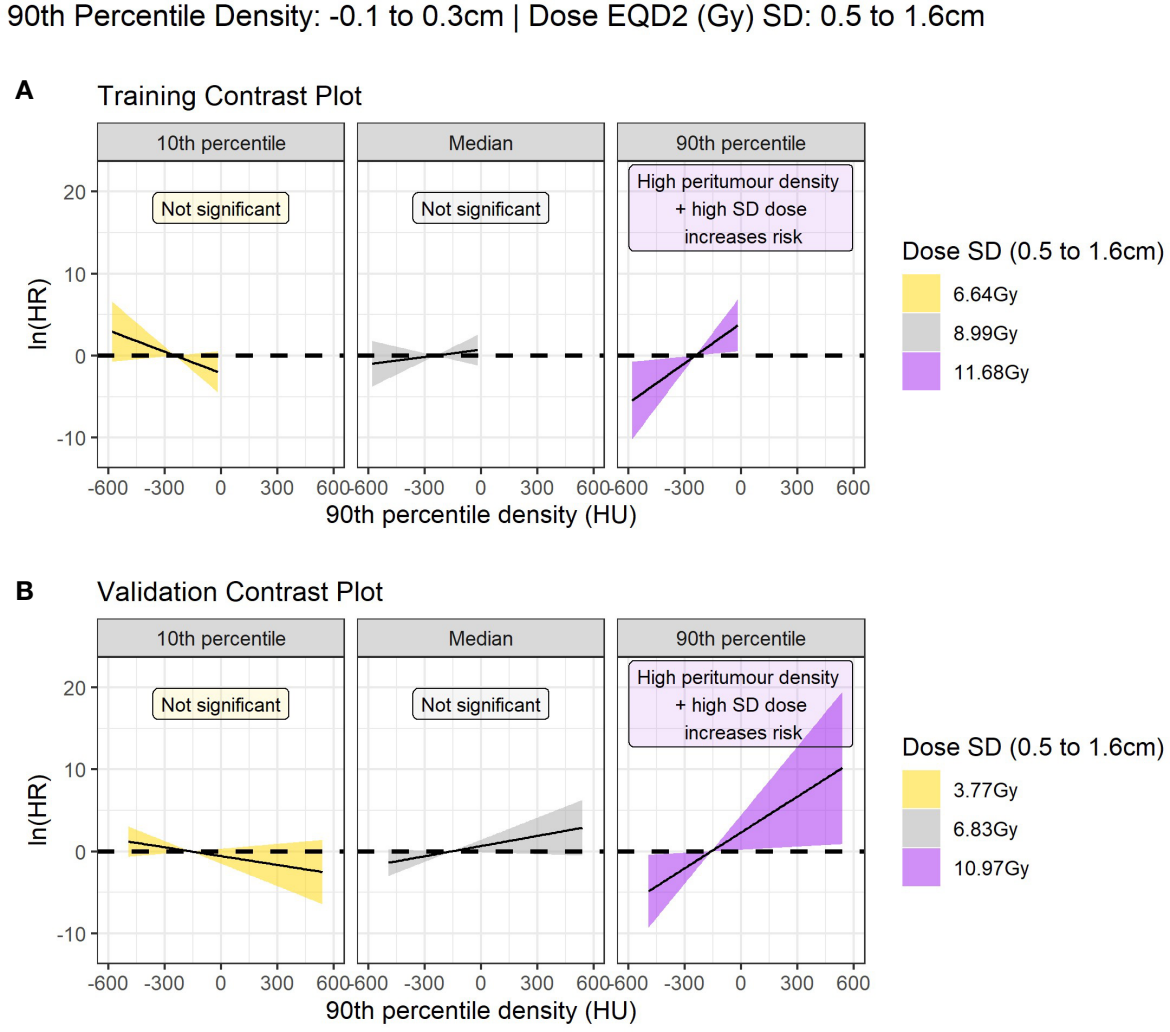
Contrast plot displaying the log(hazard ratio) versus 90^th^ percentile peritumour density at different values of dose SD (standard deviation). Significant association between density and dose is only detected at high dose variability. In this case, higher peritumour density is associated with increased risk. **(A)** Shows the results in the training data, and **(B)** shows the validation results.

**Figure 4:**
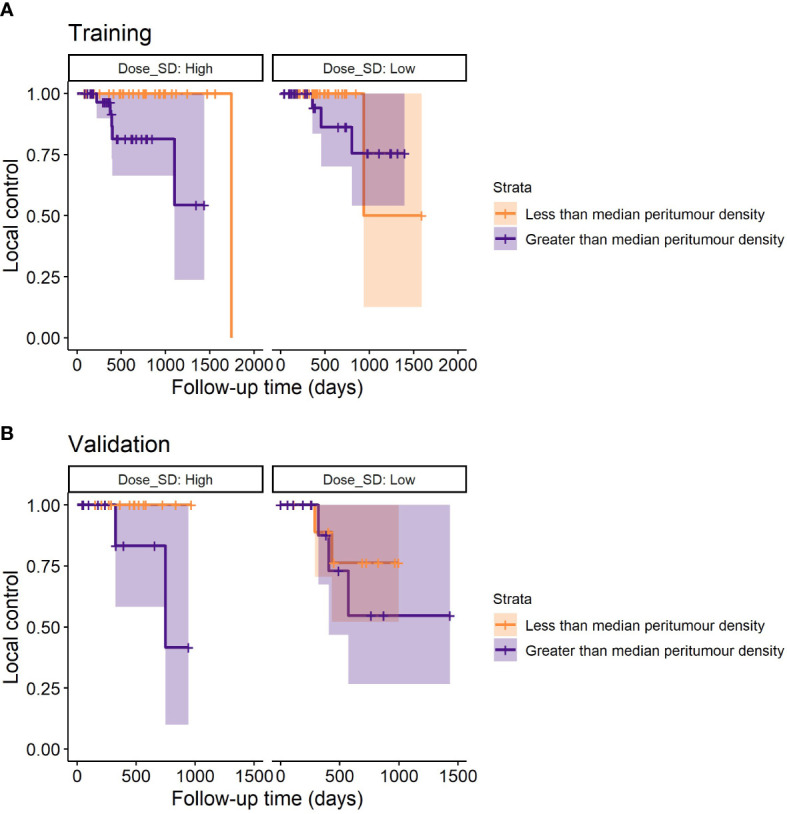
Example stratification of LR based on the median peritumour density for the lowest and highest third of dose SD *(discarding uninformative middle values)*. For low dose SD (assumed adequate MDE coverage), peritumour density no longer stratifies patients for LR – suggesting that peritumour density is a potential predictor for MDE. **(A)** Shows the survival plots on the training data, and **(B)** shows the validation data.

For RF, the maps before and after post-processing are shown in SM, **Figures 3** and **4** where between 25-100% of spurious significant pixels were removed across maps. Two regions remained (SM, **Table 4**), but neither met the 3mm threshold for annuli size and hence no regions were considered for further analysis of regional failure.

**Table 4:** The hazard ratios (HR) and p-values for the final models built in the training and validation data for predicting LR.

	Training		Validation	
	HR	P-value	HR	P-value
**ln(Tumour volume)**	4.29	**0.008**	0.53	0.095
**Tumour motion [cm]**	1.68	0.334	1.22	0.784
**Tumour location (lower lobe reference)**	10.3	**0.047**	0.52	0.454
**Age [years]**	1.01	0.885	0.97	0.552
**Sex (female reference)**	1.4	0.619	8.36	0.059
**90th percentile density -0.1 to 0.3cm from GTV [unit: 100HU]**	0.02	**0.013**	0.27	**0.057**
**Dose variability 0.5 to 1.6cm from GTV [Gy]**	2.90	**0.02**	1.20	0.184
90th percentile density * Dose variability [100HU * Gy]	1.64	**0.009**	1.29	**0.035**

The bold values are those that are significant predictors in the model (p<0.05).

### 3.5 Final training and validation models

The first column of **Table 3** reports the median and 95% confidence interval of the training C-index for the clinical LR model, the model with additional dosimetric and density information, and the final model with an interaction term. In the second column, we record the optimism-adjusted estimates of these values. In both cases, the interaction model out-performs the other models. The strict prediction-model validation results (without refitting) demonstrate that the interaction model does not directly translate to the external data, but the dose and density terms alone do improve on the clinical model in the validation data-set. However, when model coefficients were re-fitted, the importance of the interaction term was re-established.

### 3.6 Model interpretation

The model coefficients for the interaction model in the training data and for the re-fitted model in the validation data are shown in **Table 4**. The HRs for the interaction term are reported at the 90^th^ percentile of SD dose (11.7Gy) and peritumor density (-107.4HU) as this is a multiplicative model, the hazard can only be interpreted as a single value at specified values of the interacting variables.

For interpretation on the direction of effect we plot the log(hazard ratio) for peritumor 90^th^ percentile density as a continuous variable at three reference values of dose SD in **Figure 3**. The plot demonstrates that high peritumor density and high dose SD in the identified region is linked to increased risk of LR. At other values of dose SD there is no significant association between density and LR.

An example stratification with these models is demonstrated in **Figure 4**, where it can be seen that only at high dose SD, peritumour density is linked to worse outcome in training and validation cohorts.

### 3.7 Dosimetric correlations

**Figure 5:**
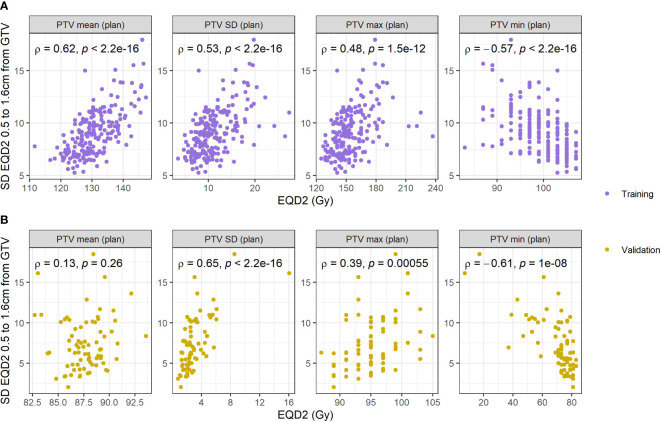
Correlations with standard deviation of the respiratory blurred dose extracted from the region identified and different dose metrics extracted from the PTV on the planned dose distribution. **(A)** Reports correlations in the training data, and **(B)** reports correlations in the validation data.

The correlations between dose in the PTV and GTV and the SD dose in the identified region was studied to investigate the relationship between *incidental* dose and more ‘standard’ dose metrics. The correlations among PTV dose metrics are shown in **Figure 5**, and among GTV metrics in SM, **Figure 5**. Dose heterogeneity (SD) in the identified region (0.5 – 1.6cm from GTV) was positively correlated with mean PTV and mean GTV dose in training data, but the correlation was weaker in the validation data. Similar observations are seen for SD, max and min dose to PTV and GTV.

## 4 Discussion

In this study based on real world datasets, as illustrated by the median age of patients and high performance status, the *`Cox-per-radius`* method developed in previous work was applied to investigate spatial interactions of density and dose for the purpose of better understanding local relapse (LR) and regional failure (RF) among early-stage lung cancer patients treated with SABR (19). We identified that higher peritumor density is significantly associated with increased chance of LR for patients who have high dose variability 0.5-1.6cm outside of the GTV. Higher dose variability in this region is likely driven by increased heterogeneity in the PTV leading to steeper dose fall-off, as shown by correlations between the PTV dose and dose in this region further out. Similarly, Salguero et al. (14) found that patients with high risk of MDE (based on higher GTV surface density and more complex shape) had increased risk of local-regional failure if receiving a low minimum dose up to 1.5cm from the GTV. Both results suggest that microscopic disease coverage is important to prevent LR and could potentially be customized according to peritumor density. Using a second real-world validation data-set, we confirmed the importance of the interaction between peritumor density and dose variability in the same location despite large demographic differences observed in the two cohorts.

Interestingly, the <1.6cm region identified is considerably narrower compared to the corresponding region discovered by our group for DM, in which dose variability and underdosage ~3cm was predominant (19). The direct mechanisms to DM, LR, and RF are not fully understood, but the idea of these being assigned to different locations of importance is supported.

No dose-density interactions were found to provide predictive ability for RF. In other work, reduced risk of RF has been linked to higher *incidental* dose to the ipsilateral hilum, which suggests RF may be a result of microscopic disease presence at the site of failure or undiagnosed nodal metastases (15). This finding could not be confirmed in this work as the radial approach removes information of proximity to specific anatomy. However, regions were identified prior to post-processing, so sensitivity testing is required to ensure we are not removing insightful information in this process. As the work of Salguero et al. reports only on local-regional failure it is impossible to determine whether it is the RF or LR that dominate their result or whether it is a combination thereof (14). The results of our study suggest considering LR and RF independently. To better understand RF, anatomical information could be included by combining density interactions with a voxel-based dose analysis (30), but this was beyond the scope of this work.

It is promising that the results of our study generalize to an external validation cohort despite differences in CT acquisition parameters. We did not apply any correction for scanner differences, since inter-scanner variability is typically small compared to inter-patient variability for density metrics (otherwise known as first-order radiomic features) (31). Differences between cohorts may require controlling for more complex texture features, such as, the Grey Level Run Length Matrix features. In this study, the focus on density features was motivated by interpretability and preliminary radiomic analysis that shown relation between 90^th^ percentile density and metastasis prediction (24). In addition to other radiomic features, different imaging modalities could be considered to improve prediction of recurrence, e.g. 18-FDG PET (32). Imaging that tracks changes during treatment (e.g., cone-beam CT) could also be utilized to consider tumor reduction (33), and the location of the surrounding microscopic disease (34).

A limitation of the methodology presented in this work is the potential risk of spurious correlations between density at the border of an automatically generated GTV and the spatially offset dose annuli. In particular, density at the peritumor border is closely linked to the ability to accurately define the GTV. Higher peritumor density could be representative of high density spiculations that may or may not be included in the GTV or demonstrate an under-sampled iGTV for tumors with large motion amplitude (35, 36). Such contour variation could link to under-treatment of the GTV as opposed to microscopic disease. In addition, an organ-at-risk abutting the GTV could be associated with an increase in peritumor density. Although care was taken to visually check all contours this cannot be fully excluded as a potential confounding factor. The influence of these factors on the association between high peritumor density and LR could be further studied by exploring the impact of contour variation on LR (37). Further, adopting a physics-approach of annuli at set distances regardless of surrounding anatomy means the dose annuli assessed in this study are not restricted to specific anatomical locations. The annuli can, therefore, include regions for some patients where microscopic disease is unlikely to be a biologically plausible route to LR (i.e., chest-wall) (14). To investigate the sensitivity of the method, a further improvement could include using dose information sampled from the lungs only (similar to density biomarkers), but one would have to be cautious as this could also lead to bias due to loss of different amounts of data when performing lung cropping for peripheral tumors or those close to surrounding organs. The radial data-mining technique demonstrated may provide information on routes to LR, but it would be important to assess histological characteristics of the tumor when making biological conclusions. This was limited by access to such information in routine practice.

There was also limited access to information on baseline pathologies that are common in lung cancer patients (e.g., chronic obstructive pulmonary disease (COPD) and emphysema) that have CT density presentations (e.g., fibrosis and bullae) that could make SABR planning more difficult. Such pathologies could influence the dose-density analysis, so care was taken to review images, dose, and density curves to detect possible outliers. In future study, this information such be incorporated in the analysis to understand causal links behind the associations identified in this work. Another potential avenue of further investigation could involve the underlying reason of the importance of dose alone (13, 38), while in this study and others the dose and density interaction is required to identify a similar association with outcomes (14, 19). This may in part be due to cohort differences, including tumor size or planning techniques combined with typically small cohort sizes and low number of events all contributing to limited power to determine true effects. Despite the low event rates, we were able to both produce a predictive model for LR with an optimism-adjusted performance of 0.82 in two independent data sets. In particular, the two cohorts included difference in demographics (i.e., performance status), tumor volume, and tumor location, which is representative of the challenges faced using real-world data as opposed to carefully selected patients enrolled on a clinical trial. Despite these challenges, we identified significant support towards the peritumor density as a predictive image biomarker, and increased dose variability up to 1.6cm for high-risk patients leads to worse clinical outcomes. The larger SD in density outside of the GTV in the validation cohort suggests that GTV delineations are tighter, which is consistent with the higher observed LR rate.

Furthermore, the higher LR rate observed in the validation data could also be explained by the lower overall dose (50Gy in 5 compared to 60Gy in 5). Here it is relevant to note that the model of Jeong et al., available online at https://tcp4rt.info, which predicts local failure rates quite close to those observed (39). Namely: predicted failure rates: 6.3% (12Gy x 5), 13% (10Gy x 5); observed rates: 6.7% (12Gy x 5), 16% (10Gy x 5). The Jeong fit to early-stage lung cancer SBRT saturates at a failure rate of 5%, even for very high biological effective dose. It seems reasonable to hypothesize that the mechanism identified in this paper, namely, underdosing of peripheral local disease, is partly responsible for these commonly observed high-dose failures.

The interaction between peritumoral density and dose variability to the identified region (0.5 – 1.6cm from GTV) maintained significance in external validation despite differences in planning approaches implemented at both institutions. The difference in planning approaches was demonstrated by difference in dosimetric correlations between the two cohorts. In training data, a positive correlation was found between standard deviation of dose to identified region, and mean PTV and mean GTV dose. This correlation was weaker in the validation data. In future, it would be worth comparing planning approaches to determine how to best homogenize dose to the identified region.

The inclusion of interaction terms in analysis of real-world data-sets has so far been under investigated. Whilst the current gold-standard evidence for a predictive biomarker is a significant *‘interaction test’* in a randomized controlled trial (40), we have demonstrated inclusion of dose-density interactions in retrospective analyses could allow us to explore and hypothesize on the impact of personalized radiotherapy using real-world data-sets. As randomized controlled trials have long timescales and are limited to specific populations, this complementary method is beneficial to assess the impact of smaller changes to clinical practice in a real-world cohort (41). The discovery and validation of a significant interaction suggests that patients could potentially be stratified based on risk of local relapse pre-treatment which could lead to changes in radiotherapy delivery (e.g., increased margins or dose intensification) or selection for immunotherapy to improve patient outcome.

## 5 Conclusion

In summary, we have applied a previously described data-mining technique to predict LR and RF following lung SABR. High peritumor density was found to interact with dose variability up to 1.6cm outside the GTV and they jointly predicted LR in two independent data-sets from different institutions. No direct association with clinical outcome was found in GTV and PTV dose metrics, but correlations demonstrated that within PTV heterogeneity may be chiefly responsible for this interaction. Overall, external confirmation of the model supports the use of density biomarkers to predict risk of microscopic disease extension, and that adequate dose coverage outside the intended treatment region of the tumor could reduce the risk of local failure. The proposed density x dose biomarker could be investigated in the future to personalize SABR dose distributions and possibly to reduce the rate of residual failures observed even at high doses.

## Data availability statement

The datasets presented in this article are not readily available because ethical permission was not granted for general publication of the dataset. Requests to access the datasets should be directed to Dr Alan McWilliam, alan.mcwilliam@manchester.ac.uk and Dr Maria Thor, thorm@mskcc.org.

## Ethics statement

Our retrospective analysis of anonymized routine data was approved by institutional information governance and research ethics committee (The Christie NHS Foundation Trust and Caldicott Committee). The research was carried out according to a protocol approved by the Caldicott committee. A data-sharing agreement was in-place for remote analysis of the data from Memorial Sloan Kettering Cancer Center.

## Author contributions

AD developed the methodology, collated the data, performed primary analysis, and wrote the manuscript. MT collated the validation data and was involved in detailed discussions about the planned validation work. AM and MH provided expert insight at all stages of the experiment. CF-F provided expert clinical insight to the project. AR and JD provided insight into the presentation of results and supported the validation work. All authors contributed to the article and approved the submitted version.
