# Supplementary material for: Predicting cancer relapse following lung stereotactic radiotherapy: an external validation study using real-world evidence
Source: Front Oncol. 2023 Jul 12;13:1156389. doi: 10.3389/fonc.2023.1156389 (PMC10369005; doi:10.3389/fonc.2023.1156389)
Supplement: Supplementary file 1 [file DataSheet_1.docx]

Supplementary Material

# Training Data

The clinical imaging data collected for training consisted of 4D-CT scans that were acquired in routine clinical practice using Philips Brilliance-CT Big Bore Oncology and Philips Bellows Device to measure respiratory signal, with the following standard acquisition parameters for all patients: 120kVp, 800mAs and use of contrast enhancement. All scans had pixel size of 3x1x1mm.

For dosimetric analysis, all plans were extracted from Philips Pinnacle treatment planning system. Prescription was planned to the D95 PTV, and the collapsed cone algorithm (*Type B*) was used for calculation with a 2mm deposition grid on the average intensity projection of the 4D-CT.

# Validation Data

For the clinical imaging data collected for validation, 4D-CT scans were acquired in routine practice with either Philips Brilliance-CT Big Bore Oncology or Discovery ST GE Medical systems. With 120 or 140kVP respectively. Scans had a voxel size of either 2x1x1mm or 2.5x1x1mm. For dosimetric analysis, all plans were extracted from ARIA RadOnc Varian Eclipse planning system. Prescription was planned to the 100% isodose line surrounding the PTV, and the Eclipse AAA algorithm was used.

Table 1 demonstrates the range of fractionation regimes implemented in the overall validation cohort.

| Prescription Dose (Gy) | No. of fractions | No. of patients |
| --- | --- | --- |
| 50 | 5 | 95 |
| 60 | 8 | 16 |
| 60 | 15 | 10 |
| 48 | 4 | 8 |
| 45 | 5 | 5 |
| 54 | 3 | 4 |
| 46 | 5 | 1 |

Table 1. The dose prescription regimes used in validation dataset. The number of patients are the number within the cohort on that particular regime.

# Training clinical models

Table 2 reports on the full multivariable Cox model results for the clinical data only in predicting regional failure and local relapse.

|  | **Regional** | | **Local** | |
| --- | --- | --- | --- | --- |
|  | **HR (95% CI)** | **P-value** | **HR (95% CI)** | **P-value** |
| **ln(Tumour volume)** | 1.67 (0.97-2.89) | 0.066 | 3.05 (1.39-6.72) | **0.006** |
| **Motion amplitude (cm)** | 1.73 (0.79-3.75) | 0.168 | 2.52 (0.96-6.59) | 0.060 |
| **Tumour location (lower reference)** | 0.76 (0.22-2.58) | 0.660 | 3.70 (0.59-23.1) | 0.162 |
| **Age (years)** | 0.96 (0.91-1.02) | 0.198 | 0.98 (0.92-1.05) | 0.601 |
| **Sex (female reference)** | 1.16 (0.41-3.25) | 0.784 | 1.56 (0.47-5.22) | 0.472 |

Table 2. Clinical multivariable model for to predict regional failure and local relapse. No variables are significantly associated with outcome for regional (left), and tumour volume is for local failure (right).

Table 3 demonstrates the updated Akaike Information Criterion (AIC) of the clinical regional failure and local relapse Cox models based on the inclusion of ‘standard’ dose metrics. As demonstrated by the p-value of the likelihood-ratio test between the clinical model and the clinical plus dose model there is no improvement in prediction from dose metrics.

| **Dose metric** | **Regional** | | **Local** | |
| --- | --- | --- | --- | --- |
|  | **AIC** | **P value** | **AIC** | **P value** |
| Mean GTV (blurred) | 144.2 | 0.743 | 101.4 | 0.440 |
| SD GTV (blurred) | 144.3 | 0.926 | 101.3 | 0.410 |
| Max GTV (blurred) | 144.3 | 0.840 | 101.9 | 0.705 |
| Min GTV (blurred) | 144.3 | 0.935 | 99.3 | 0.100 |
| Mean PTV (plan) | 143.9 | 0.504 | 101.7 | 0.583 |
| SD PTV (plan) | 144.3 | 0.837 | 101.9 | 0.790 |
| Max PTV (plan) | 144.3 | 0.851 | 101.9 | 0.750 |
| Min PTV (plan) | 143.9 | 0.497 | 101.8 | 0.653 |

Table 3. AIC and result of the likelihood-ratio test from including the dose metric in the model reported in Table 1. No dose parameters are statistically significant for either outcome.

# Local relapse results

**
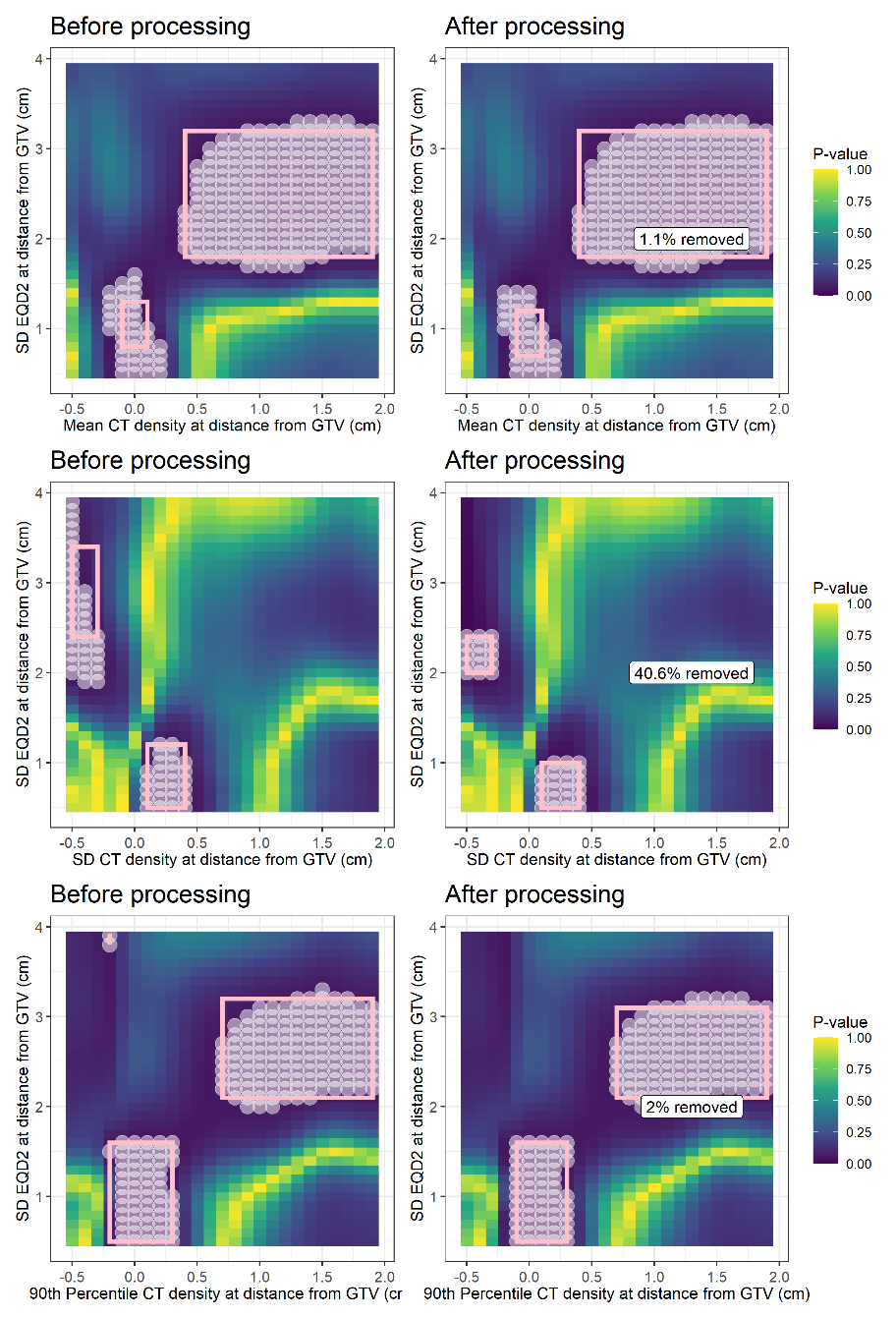
**Figure 1 demonstrates the heat-map results for the Cox-per-radius analysis of local relapse considering dose standard deviation, before and after post-processing was applied.

Figure 1. Cox-per-radius significance map of interaction between standard deviation EQD2 at distance from the GTV (y-axis) with from top to bottom: mean, standard deviation and 90^th^ percentile density (x-axis) to predict local relapse. Before (left) and after (right) size post-processing is displayed.


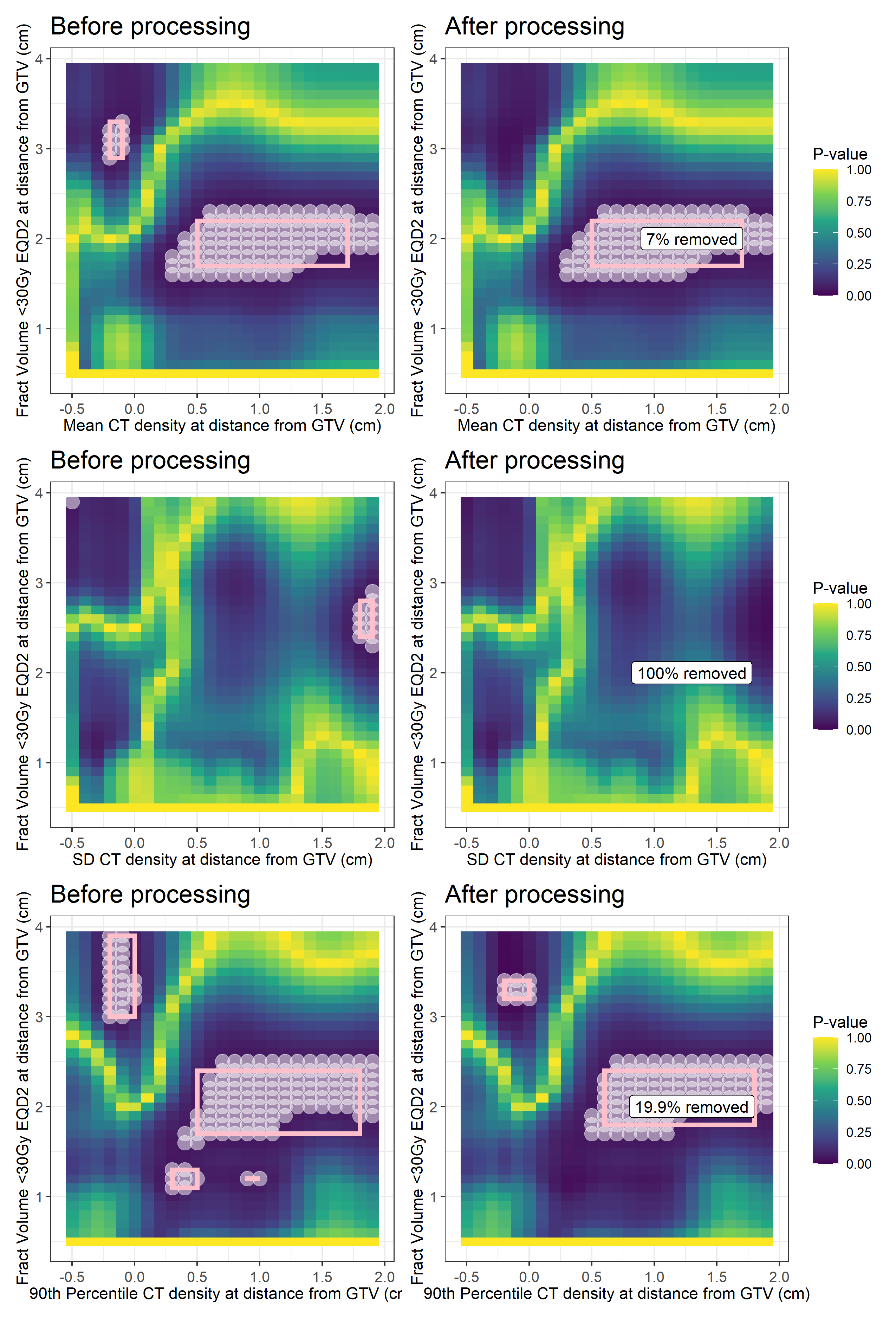
Figure 2 demonstrates the heat-map results for the Cox-per-radius analysis of local relapse considering fraction of volume receiving less than 30Gy EQD2, before and after post-processing was applied.

**Figure 2.** Cox-per-radius significance map of interaction between fraction of volume receiving less than 30Gy EQD2 at distance from the GTV (y-axis) with from top to bottom: mean, standard deviation and 90^th^ percentile density (x-axis) to predict local relapse. Before (left) and after (right) size post-processing is displayed.

After this analysis, nine candidate regions were identified on each map, and the analysis of these regions is reported in Table 3. Three were below the size threshold (purple), one had unstable coefficients on bootstrap resampling (blue) and four did not improve on the clinical model performance (orange), leaving only one for further analysis.

| Density metric | Dose metric | Density region | Dose region | Density median (range) | Dose median (range) | C-index median (95% CI) | Freq | Coef (95% CI) |
| --- | --- | --- | --- | --- | --- | --- | --- | --- |
| **90th percentile** | **Standard deviation** | **-0.1 to 0.3** | **0.5 to 1.6** | **-241 (-580 –**  **-16)** | **8.99 (5.27 - 17.92)** | **0.81 (0.7-0.85)** | **372** | **0.02-0.29** |
| Mean | Standard deviation | -0.1 to 0.1 | 0.7 to 1.2 | -332 (-716 –  -122) | 8.33 (4.55 - 17.27) | 0.81 (0.69-0.85) | 364 | 0-0.24 |
| 90th percentile | Fract vol <30Gy | -0.2 to 0.0 | 3.2 to 3.4 | -38 (-421 - 294) | 0.79 (0.36 - 0.99) | 0.83 (0.73-0.86) | 363 | -5.97--0.71 |
| Standard deviation | Standard deviation | -0.5 to -0.3 | 2.0 to 2.4 | 59 (14 - 302) | 13.26 (6.08 - 19.5) | 0.81 (0.68-0.85) | 335 | -0.17-0 |
| Mean | Standard deviation | 0.4 to 1.9 | 1.8 to 3.2 | -780 (-940 –  -443) | 12.71 (6.5 - 18.54) | 0.78 (0.65-0.82) | 294 | 0.02-0.32 |
| Standard deviation | Standard deviation | 0.1 to 0.4 | 0.5 to 1.0 | 148 (61 - 236) | 7.41 (3.88 - 17.53) | 0.78 (0.65-0.81) | 282 | 0.01-0.43 |
| 90th percentile | Standard deviation | 0.7 to 1.9 | 2.1 to 3.1 | -660 (-885 –  -84) | 12.95 (5.63 - 18.7) | 0.78 (0.64-0.82) | 275 | 0-0.16 |
| 90th percentile | Fract vol <30Gy | 0.6 to 1.8 | 1.8 to 2.4 | -654 (-884 –  -77) | 0.19 (0 - 0.46) | 0.79 (0.66-0.84) | 270 | -0.04-4.54 |
| Mean | Fract vol <30Gy | 0.5 to 1.7 | 1.7 to 2.2 | -783 (-942 –  -449) | 0.12 (0 - 0.35) | 0.78 (0.64-0.84) | 260 | 0.16-5.15 |

Table 3. Nine regions identified after post-processing (shown in in Figure 1 and 2). Those highlighted in purple were removed for being less than 3mm in size in either direction, those in blue had unstable coefficients over bootstrap resampling as the 95% confidence interval lies either side of 0, and those in orange represent a C-index which does not improve on the clinical model. The remaining region in black was selected for the rest of analysis.

# Regional failure results


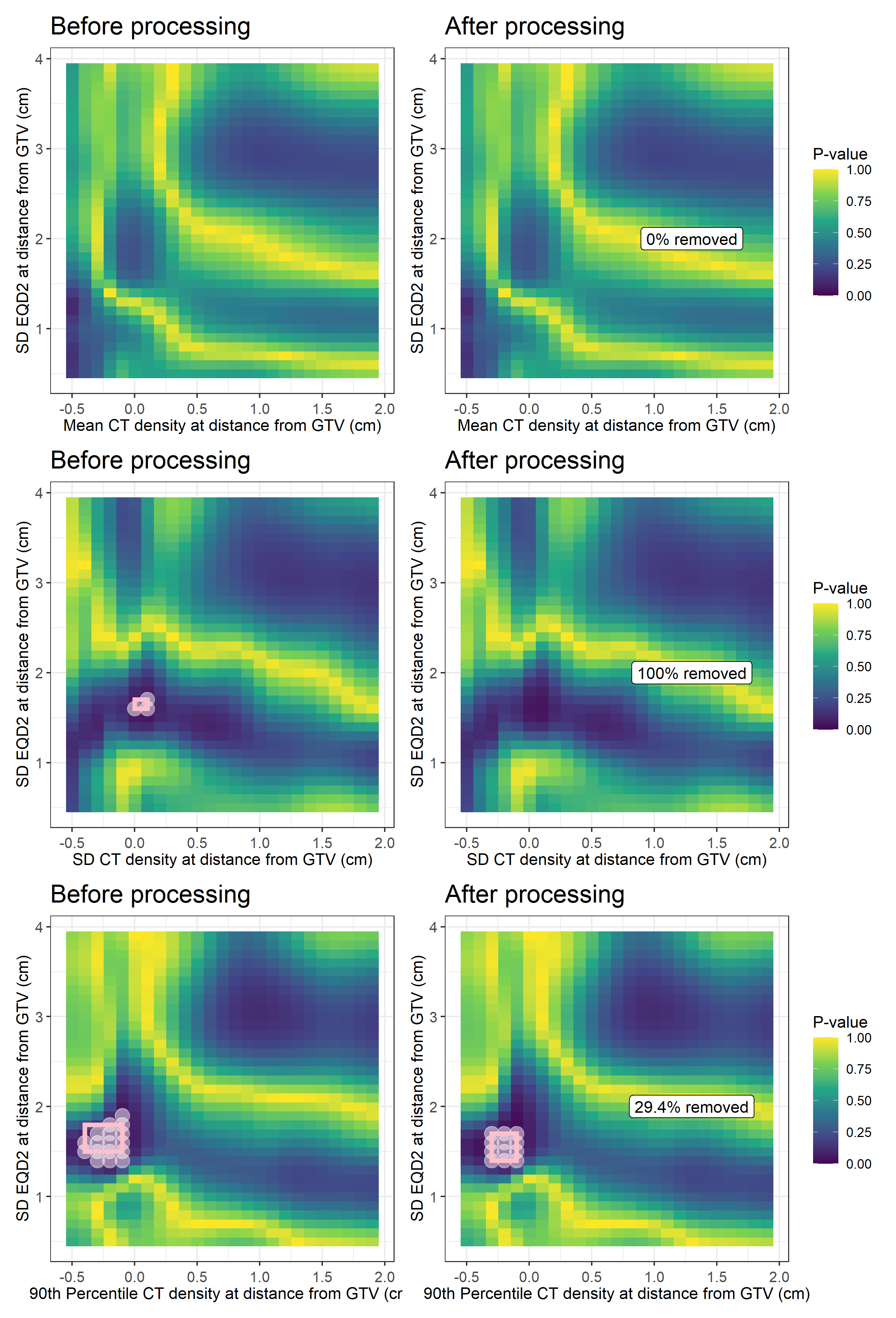
Figure 3 demonstrates the heat-map results for the Cox-per-radius analysis of regional failure considering dose standard deviation, before and after post-processing was applied.

**Figure 3.** Cox-per-radius significance map of interaction between standard deviation EQD2 dose at distance from the GTV (y-axis) with from top to bottom: mean, standard deviation and 90^th^ percentile density (x-axis) to predict regional failure. Before (left) and after (right) size post-processing is displayed.

Figure 4 demonstrates the heat-map results for the Cox-per-radius analysis of regional failure considering fraction of volume receiving less than 30Gy EQD2, before and after post-processing was applied.


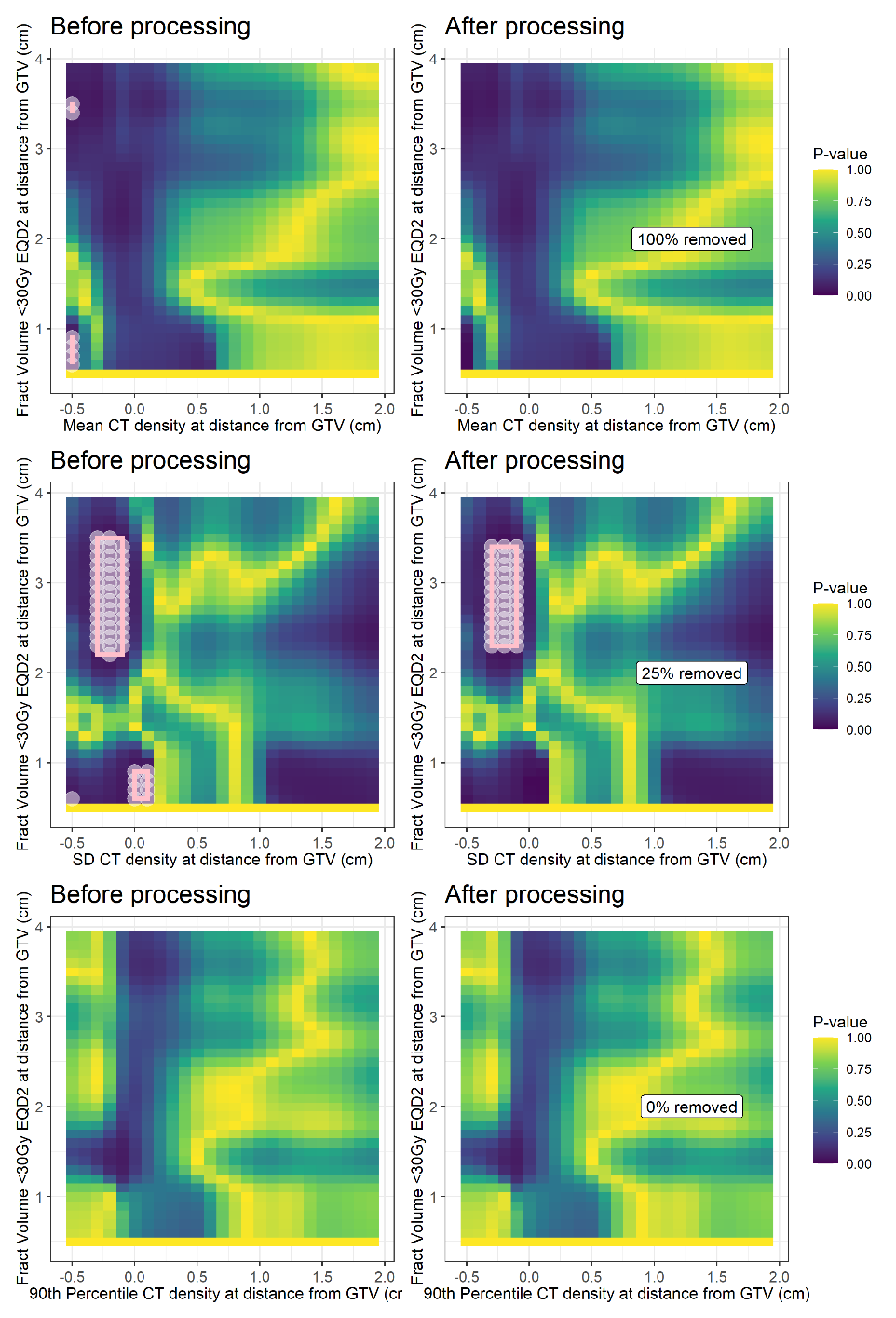


**Figure 4.** Cox-per-radius significance map of interaction between fraction of volume receiving less than 30Gy EQD2 at distance from the GTV (y-axis) with from top to bottom: mean, standard deviation and 90^th^ percentile density (x-axis) to predict regional failure. Before (left) and after (right) size post-processing is displayed.

Two candidate regions remained following the regional failure Cox-per-radius analysis, but neither met the 3mm annuli size threshold for further analysis.

| **Density metric** | **Dose metric** | **Density region** | **Dose region** | **Density median (range)** | **Dose median (range)** | **C-index** | **Freq** |
| --- | --- | --- | --- | --- | --- | --- | --- |
| Standard deviation | Fract vol <30Gy | -0.3 to  -0.1 | 2.3 to 3.4 | 111 (40 - 254) | 0.59 (0.18 - 0.86) | 0.79 (0.7-0.83) | 464 |
| 90th percentile | Standard deviation | -0.3 to  -0.1 | 1.4 to 1.7 | 21 (-292 - 572) | 11.1 (6.18 - 21.0) | 0.72 (0.6-0.78) | 275 |

Table 4. Two regions identified after post-processing (shown in in Figure 3 and 4) were deemed too small to be of significant value.

# Dosimetric correlations

Figure 5 displays correlations between *‘incidental dose’* standard deviation in the region identified and more ‘standard’ dose metrics extracted from the GTV.


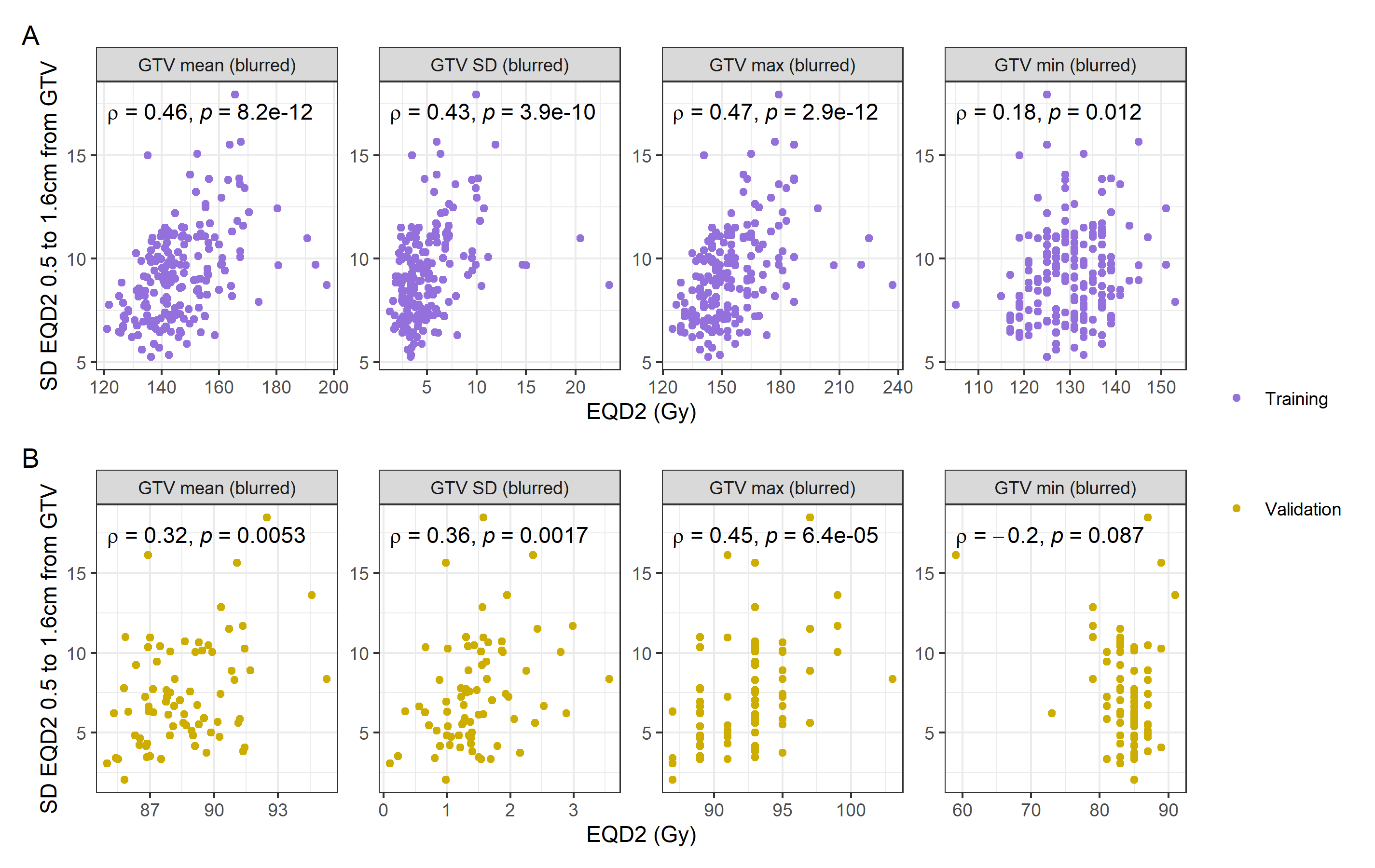


**Figure 5.** Correlations with standard deviation of the respiratory blurred dose extracted from the region identified and different dose metrics extracted from the GTV on the blurred dose distribution.
